# Coordinated and Independent Roles for MLH Subunits in DNA Repair

**DOI:** 10.3390/cells10040948

**Published:** 2021-04-20

**Authors:** Gianno Pannafino, Eric Alani

**Affiliations:** Department of Molecular Biology and Genetics, Cornell University, Ithaca, NY 14853-2703, USA; gnp6@cornell.edu

**Keywords:** MutL homologs, MLH, DNA mismatch repair, meiosis, Holliday junction resolution, homologous recombination

## Abstract

The MutL family of DNA mismatch repair proteins (MMR) acts to maintain genomic integrity in somatic and meiotic cells. In baker’s yeast, the MutL homolog (MLH) MMR proteins form three heterodimeric complexes, MLH1-PMS1, MLH1-MLH2, and MLH1-MLH3. The recent discovery of human PMS2 (homolog of baker’s yeast PMS1) and MLH3 acting independently of human MLH1 in the repair of somatic double-strand breaks questions the assumption that MLH1 is an obligate subunit for MLH function. Here we provide a summary of the canonical roles for MLH factors in DNA genomic maintenance and in meiotic crossover. We then present the phenotypes of cells lacking specific MLH subunits, particularly in meiotic recombination, and based on this analysis, propose a model for an independent early role for MLH3 in meiosis to promote the accurate segregation of homologous chromosomes in the meiosis I division.

## 1. Introduction

**Figure 1:**
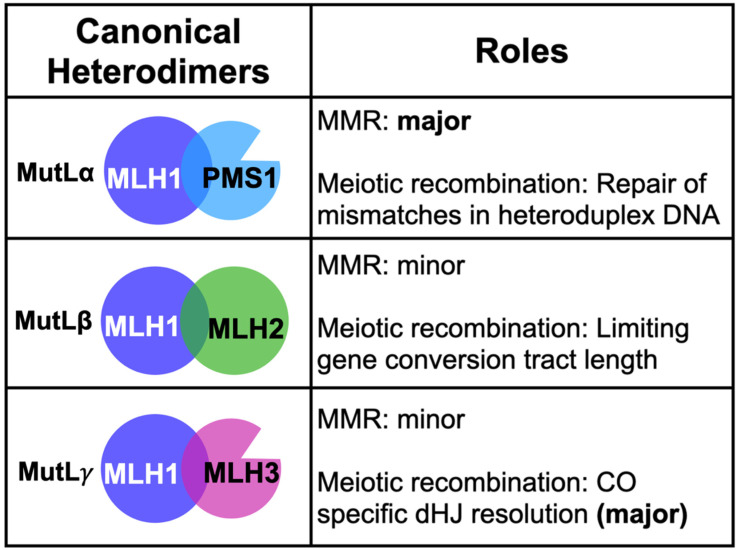
Summary of roles for canonical MLH complexes in MMR and meiotic recombination (see text for details).

The eukaryotic MutL homolog (MLH) family of DNA mismatch repair (MMR) proteins consists of three heterodimeric complexes conserved from baker’s yeast to humans. In baker’s yeast, they are referred to as MLH1-PMS1 (MutLα), MLH1-MLH2 (MutLβ), and MLH1-MLH3 (MutLγ) (Figure 1). In this review, we use by default the baker’s yeast designations for the MLH factors, and when appropriate, specify the mammalian designations. As explained more fully below, MLH proteins act in both MMR and meiotic recombination; MLH1-PMS1 functions as the primary MLH factor in MMR with MLH1-MLH2 and MLH1-MLH3 playing minor roles, though MLH1-MLH3 has been implicated in trinucleotide repeat expansion steps that appear related to MMR. In meiotic recombination, MLH1-MLH2 limits the length of heteroduplex tracts that arise from strand invasion events that are initiated from double-strand breaks, and MLH1-MLH3 is critical for the faithful segregation of homologous chromosomes in meiosis I division by ensuring crossover-specific resolution of double Holliday junctions [1,2,3,4].

Phylogenetic studies provided evidence for the occurrence of ancient gene duplication events that led to the specialization of the MLH family of paralogs. Such work also indicated that the MLH1 outgroup diverged prior to the splits that led to the PMS1, MLH2, and MLH3 paralogs [5,6,7,8]. One of the long-lasting paradigms of the eukaryotic MLH family is MLH1 serving as a common partner for PMS1, MLH2, and MLH3 (Figure 1). In Section 2, we present the well-established MLH1-dependent roles in three cellular contexts: DNA MMR, trinucleotide repeat expansion, and meiotic recombination. In Section 3, we examine recent evidence hinting at MLH1-independent roles for MLH3 in homologous recombination. Lastly, in Section 4 we consolidate these observations into a model proposing that in meiosis, MLH3 plays an early, MLH1-independent role to stabilize nascent joint molecules that form during initial stages of recombination.

## 2. Coordinated Roles for the MLH Proteins in DNA Metabolism

To explore new roles for the MLH family in meiosis, we first present the established roles for this family in various cellular contexts. Section 2 then outlines studies showing that MLH1 is a critical component of heterodimeric MLH complexes that act in a variety of DNA-repair events.

### 2.1. MLH Family Proteins Function Together in Post-Replicative DNA-Mismatch Repair

MMR enforces genomic integrity by repairing misincorporated nucleotides and loops resulting from DNA-slippage events that are introduced during DNA replication. Much of our insights into the mechanism of MMR come from the bacterial *Escherichia coli* system. In *E. coli*, mismatch recognition by the MutS homodimer triggers ATP-dependent conformational changes, which convert MutS into a sliding clamp capable of recruiting the MutL homodimer. The MutS-MutL complex then recruits the MutH endonuclease to nick the newly replicated unmethylated strand in a process temporally regulated by the activity of DNA adenine methyltransferase (Dam). The nicked DNA serves as an entry site for the unwinding of DNA by the UvrD helicase. Unwound single-stranded DNA is then digested by exonucleases to create a gap through the mismatch site that is repaired by DNA polymerase and ligase ([9,10] and reviewed in [1,11]).

**Figure 2:**
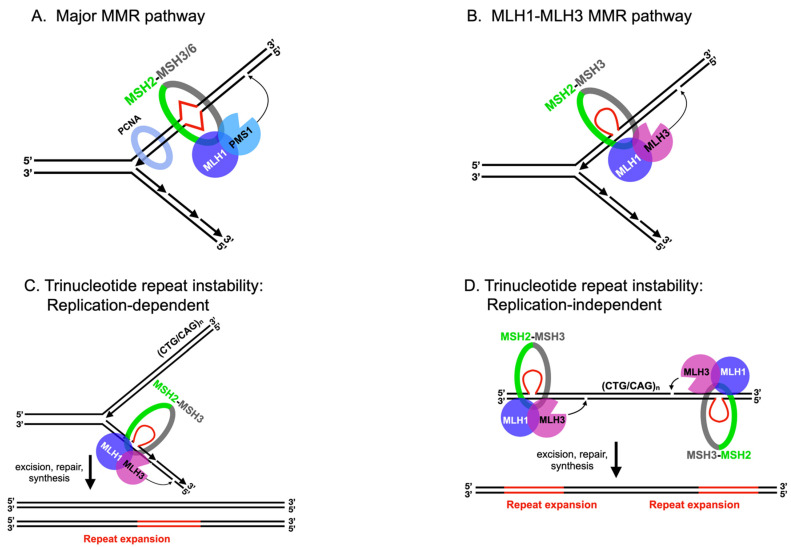
Roles for MLH complexes in DNA mismatch repair and trinucleotide repeat instability. (**A**) MLH1-PMS1 plays a major role in MMR. Upon recruitment to the site of a mismatch by either MSH2-MSH6 or MSH2-MSH3, MLH1-PMS1 is directed by PCNA to nick the newly synthesized strand enabling downstream factors to excise the mismatch and resynthesize the excised DNA. (**B**) MLH1-MLH3 plays a minor role in MMR, where it is recruited to deletion-loop mismatches by MSH2-MSH3 and is stimulated to nick the strand opposite a deletion loop. Restoration of the template sequence proceeds as described for MLH1-PMS1. (**C**) MLH1-MLH3 acts in trinucleotide repeat expansion during DNA replication. In this version of a model for trinucleotide repeat expansion, MLH1-MLH3 nicks the strand opposite a DNA loop formed in the lagging strand in a reaction dependent on loop recognition by MSH2-MSH3. (**D**) MLH1-MLH3 acts in trinucleotide repeat expansion independent of DNA replication [33]. As in (**C**), MLH1-MLH3 nicks the strand opposite DNA loops formed in trinucleotide repeat sequences in a reaction dependent on loop recognition by MSH2-MSH3.

In eukaryotes, mismatches and insertion/deletion loops are recognized by MutS homolog (MSH) heterodimers. MSH2-MSH6 primarily recognizes base–base mismatches and 1 nt insertion/deletion loops, and MSH2-MSH3 primarily recognizes insertion/deletion loops that can be as large as 17 nt in size [1,12,13,14] (Figure 2A). Recent models propose that mismatch recognition by MSH heterodimers triggers ATP-dependent conformational changes in these proteins, converting them into sliding clamps that primarily recruit MLH1-PMS1, which contains an endonuclease motif within the PMS1 subunit. These steps result in MLH1-PMS1 nicking the newly replicated strand through interactions with PCNA, enabling downstream factors to act through multiple pathways to excise the misincorporated DNA. The resulting gap is resynthesized by DNA polymerases and sealed by DNA ligase ([15,16] and reviewed in [1,17]). MLH1-MLH2 and MLH1-MLH3 are also recruited to mismatches by MSH heterodimers, but their roles appear to be narrower than for MLH1-PMS1, as indicated by the relatively minor defects in MMR seen in yeast in *mlh2Δ* and *mlh3Δ* mutants [7,18,19,20,21].

Numerous in vitro biochemical and genetic studies in MMR have suggested a requirement for MLH1 as a subunit of all three heterodimers. All four eukaryotic MLH proteins possess an N-terminal ATP binding domain, an unstructured linker, and a structured C-terminal domain. The asymmetric binding of ATP to MLH1 and its MLH partner triggers conformational changes that facilitate the coordinated condensation of the N-terminal domains of the complex, bringing them in close proximity to the C-termini [1,22,23]. Accordingly, MLH1 exhibits higher affinity for ATP than PMS1 [24], and mutations in MLH1′s ATPase motifs confer greater MMR defects than mutations in PMS1′s ATPase motifs [25]. Given their structural similarities, we infer that MLH1-MLH2 and MLH1-MLH3 undergo ATP-dependent conformational changes upon interacting with their substrates in mechanisms analogous to that seen for MLH1-PMS1 [23,26,27]. Furthermore, these studies provide a coherent rationale for why MLH1 is the common subunit for all known MLH heterodimers.

Although MLH1-PMS1, MLH1-MLH2, and MLH1-MLH3 share many similarities in their modes of action during MMR, distinctions can be made between their structure, mechanisms of recruitment, substrate specificity, and strand-nicking specificity (Figure 2). First, PMS1 and MLH3 contain endonuclease motifs, whereas MLH2 does not. Consistent with these observations, MLH1-PMS1 and MLH1-MLH3 display endonuclease activities, setting them apart from *E. coli* MutL, which lacks endonuclease activity and instead relies on MutH for strand-specific nicking [21,28,29,30], though most bacteria contain MutL with an intrinsic endonuclease activity and likely use a strand discrimination mechanism more similar to eukaryotic MMR [31]. MLH1-MLH2, however, lacks such an activity [4].

Second, the variation in functionality between MLH complexes is reflected in their recruitment and substrate specificity in MMR. MLH1-PMS1 is recruited to repair base–base mismatches and insertion/deletion loops by either MSH2-MSH6 or MSH2-MSH3. MLH1-MLH2 also appears to be recruited to mismatches by both MSH2-MSH6 or MSH2-MSH3, but its exact function is less well understood, and it is considered an accessory factor [4,7]. Interestingly, MLH1-MLH3 acts in MSH2-MSH3-dependent MMR, specifically in the repair of deletion loops [19,20,21] (Figure 2B). While these observations are consistent with the hypothesis that MSH2-MSH3 directs strand-specific nicking by MLH1-MLH3, they do not rule out the possibility of an alternative in vivo strand-specificity factor. That said, the nicking activity performed by MSH2-MSH3 and MLH1-MLH3 is reminiscent of the activity exhibited by MLH1-MLH3 in other cellular contexts (discussed below for trinucleotide repeat instability) but appears distinct from the mechanism of strand specificity imposed by interactions between PCNA and MLH1-PMS1 in MMR that involve a PCNA-interacting protein-box (PIP-box) motif in PMS1 that is not found in the corresponding structural location in MLH3 [15,32].

### 2.2. MLH1-PMS1 and MLH1-MLH3 Promote Trinucleotide Repeat Expansion in Mammals

Genome-wide-association studies in human patients, as well as genetic studies in baker’s yeast, mice, and human cell cultures, have linked *MLH1, PMS1* (*PMS2* in mammals), and *MLH3* to trinucleotide repeat expansions in human diseases such as Huntington’s (CAG expansions in the *HTT* gene) and myotonic dystrophy type I (CTG/CAG expansions in the *DMPK* gene) [34,35,36,37,38,39,40,41,42,43]. Repeat expansions have been shown to occur in both germline and somatic cells through events dependent on (Figure 2C) or independent of (Figure 2D) DNA replication. Mechanistic studies in both baker’s yeast and mammals have provided models for MSH2-MSH3, MLH1-PMS1, and MLH1-MLH3 acting as mediators of small trinucleotide repeat expansions in somatic cells [34,35,37,44,45,46] (Figure 2). In these models, MSH2-MSH3 is proposed to recognize small loops within triplet repeats and recruit the MLH endonucleases MLH1-PMS1 and MLH1-MLH3.

In the context of a nonreplicating cell, repeat sequences are hypothesized to form single-stranded loops that can be excised to yield expansions or contractions following repair synthesis (Figure 2D). If the loop-containing strand is excised, DNA synthesis and ligation results in a repeat contraction. If the loop-lacking strand is excised, a repeat expansion occurs. In vitro repair assays performed by Modrich, Kadyrov, and colleagues showed that human MLH1-PMS1 is capable of generating roughly equal mixtures of expansions and contractions following nicking and excision of a 3-bp loop-containing covalently closed DNA substrate [37,45]. In contrast, human MLH1-MLH3 nicking was heavily biased toward the generation of repeat expansions in a MSH2-MSH3-dependent manner, and this bias was contingent on MLH3′s endonuclease activity. These observations indicate that in vitro reactions containing MSH2-MSH3 and MLH1-MLH3 and essential cofactors were sufficient to create the biased nicking of the loop-lacking strand of a plasmid substrate [37]. As mentioned above, MLH1-MLH3′s role in MMR is biased toward a MSH2-MSH3 repair pathway that involves the repair of deletion loops [19,20,33,46] (Figure 2B). Thus, it is hypothesized that during repeat expansion, MSH2-MSH3 and MLH1-MLH3 inappropriately identify these substrates as deletion loops (Figure 2C,D). In the context of meiosis, the ability of MLH1-MLH3 to be directed to nick the strand opposite a DNA loop may provide an attractive model for how biased cleavage of a double Holliday junction occurs (see below).

### 2.3. Roles for MLH1-MLH3 and MLH1-MLH2 in Meiotic Recombination

Meiosis is the process by which sexually reproducing eukaryotes create four haploid cells from a single diploid cell via one round of DNA replication, followed by two consecutive rounds of cell division culminating in the production of four genetically distinct haploid cells [47,48,49,50]. Crossing over denotes the reciprocal exchange of chromosome arms between synapsed homologous chromosomes. In addition to its contribution to genetic diversity by creating unique paternal/maternal hybrid chromosomes, crossing over and distal sister chromatid cohesion ensure the faithful segregation of homologous chromosomes towards opposite poles by providing the necessary tension to biorient homologs on the meiosis I spindle. Failure to receive at least one crossover (CO) per homolog pair is thought to result in homolog nondisjunction at the first meiotic division due to achiasmate homolog pairs lacking the stable physical linkage provided by a CO and distal sister chromatid cohesion. Given the crucial nature of crossing over, it is unsurprising that in humans, dysregulation of CO formation is linked to aneuploidy-associated disorders such as Down syndrome, miscarriage, and infertility [49,51].

**Figure 3:**
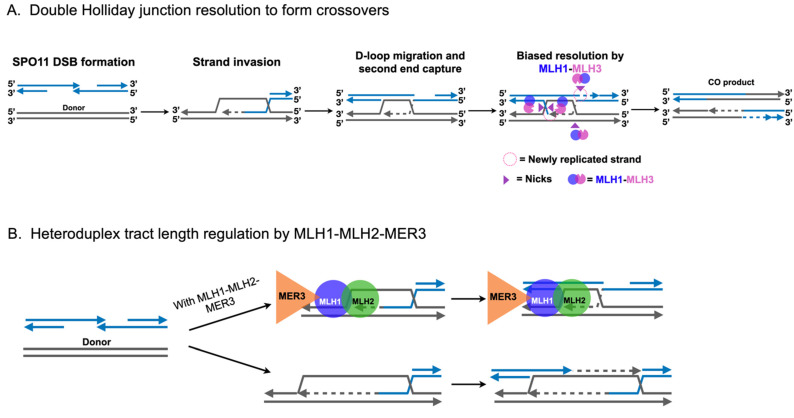
MLH1-MLH3 and MLH1-MLH2 play important roles in meiotic recombination. (**A**) MLH1-MLH3 plays a major role in meiotic crossover formation. Meiotic recombination is initiated by the formation of programmed double-strand breaks catalyzed by SPO11. Following 5′-3′ resection of break ends, the DMC1 and RAD51 recombinases form a filament on 3′ single-stranded tails, catalyzing homology search for the allelic locus on the homologous chromosome. Strand invasion is accompanied by D-loop migration and capture of the second end of the double-strand break. Ultimately, MLH1-MLH3 is recruited to ZMM-stabilized (not shown) double Holliday junctions, where it shows a bias for a nicking orientation overlapping with new DNA synthesis tracts, resulting in a crossover product. (**B**) MLH1-MLH2, in connection with the MER3 helicase, limits the length of heteroduplex tracts arising from extension of the 3′ tail of an invading double-strand break. See text for details.

In *S. cerevisiae* and higher eukaryotes, recombination leading to crossing over is initiated in meiotic prophase with the formation of SPO11 induced double-strand breaks that occur genomewide [52,53,54,55,56] (Figure 3A). These double-strand breaks undergo 5′-3′ resection to form 3′ single-stranded tails, which then undergo a homology search with a preference for repair off of the homologous chromosome as opposed to the sister chromatid. Strand invasion of the homologous donor chromosome results in the formation of a displacement loop (D-loop) in a reaction catalyzed by recombinase machinery that includes DMC1, RAD51, RAD52, and RAD54 [57,58]. Once formed, and following extension by DNA synthesis, the D-loop may be unwound by the helicase/topoisomerase STR complex (composed of SGS1-TOP3-RMI1 in baker’s yeast or BLM-TOP3-RMI1 in mammals). Such unwound intermediates can participate in subsequent strand invasions or be repaired via a synthesis-dependent strand-annealing mechanism to result in a noncrossover (NCO) product (reviewed in [59]). Alternatively, the D-loop intermediate can be extended by DNA synthesis followed by second-end capture of the other end of the double-strand break to form double Holliday junction intermediates that are resolved into crossovers. MLH1-MLH2 has been shown in genetic and biochemical studies to interact with the MER3 helicase to prevent excessive D-loop extensions and DNA synthesis (Figure 3B). Such a mechanism modulates meiotic recombination tract length, and has been suggested to limit the availability of DNA sequences that could participate in recombination events that lead to genome rearrangements [3,4]. Curiously, MLH1 interacts with SGS1 helicase in budding yeast and BLM helicase in human cells, though the relevance for such an interaction in meiotic recombination remains unclear [60,61,62].

In the major CO pathway, extended D-loops are stabilized by the meiosis-specific MSH family complex MSH4-MSH5 along with other members of the functionally diverse ZMM family, including ZIP1-4, MER3, and SPO16 (reviewed in [63]). This stabilization promotes the formation of double Holliday junctions; MLH1-MLH3 is thought to be recruited to ZMM-stabilized double Holliday junctions, where it asymmetrically cleaves them into only crossover products, potentially through interactions with PCNA [64,65,66,67] (Class I COs; Figure 3A). Importantly, the Class I CO pathway exhibits interference, which results in COs that are widely and evenly spaced, as well as CO assurance, the phenomena in which each homolog pair receives at least one CO. This pathway accounts for the 75–85% of COs in budding yeast and 90–95% of COs in mice [59,63,64].

In the Class II CO pathway (~15 to 25% of all crossovers), double Holliday junctions that arise independently of ZMMs are cleaved without orientation bias primarily by the structure-selective nuclease MUS81-MMS4 (MUS81-EME1 in mammals) to result in a roughly 50:50 mixture of CO and NCO products [59,64,68,69,70]. Additional structure-selective nucleases such as SLX1-SLX4 and YEN1 also act in this pathway as compensatory mechanisms for double Holliday junction resolution [64,71].

Numerous genetic studies in yeast and mice have indicated that both MLH1 and MLH3 are critical for meiotic CO formation [64,72,73,74]. In yeast, deletion of *MLH1* or *MLH3* results in decreases in crossing over and spore viability, as well as increases in meiosis I (MI)-nondisjunctions [2,26,75,76,77,78]. MLH3 confers the endonuclease activity of MLH1-MLH3; a mutation in the DQHA(X)_2_E(X)_4_E metal-binding motif of MLH3 (*mlh3-D523N)* abolishes the endonuclease activity of budding yeast MLH1-MLH3 in vitro, and confers *mlh3*∆-like phenotypes in vivo [21,29,78]. These and earlier data suggest that MLH1-MLH3 is responsible for resolving double Holliday junctions via the endonuclease activity of MLH3. However, MLH1-MLH3 does not conform to the paradigms of known structure-selective nucleases that are capable of recognizing Holliday junction substrates in vitro and precisely cleaving symmetrically at branch points (see examples in [79]). MLH1-MLH3 efficiently binds model Holliday junction substrates, but binding to them inhibits its endonuclease activity [21,29,30,65,66]. Rather, MLH1-MLH3 endonuclease activity is activated upon polymer formation in vitro on large double-stranded substrates [30]. It is still unclear whether MLH1-MLH3 is activated by polymer formation in vivo to cleave double Holliday junctions, but the nucleosome remodeler CHD1 has been proposed as a potential candidate for facilitating such activity [80].

An intact double Holliday junction is a symmetric molecule that must be asymmetrically cleaved by MLH1-MLH3 in the Class I pathway to yield only CO products. Recent studies have shed light on the mechanism of imparting asymmetry by analyzing heteroduplex DNA tracts following CO formation [81,82]. Using MMR-defective yeast and mice strains, respectively, Marsolier-Kergoat et al. [81] and Peterson et al. [82] observed the patterns of heteroduplex DNA recovered from MMR-defective strains. Their results delineated a model involving extensive D-loop migration in the direction of DNA synthesis following strand invasion by the broken homolog. Moreover, the patterns of heteroduplex DNA recovered suggest a strong MLH1-MLH3 dependent bias for a nicking orientation overlapping with new DNA synthesis tracts (Figure 3A). Curiously, this mode of directed nicking fits neatly with a model in which the double Holliday junction remains unligated following DNA synthesis. One hypothesis is that meiotic factors recognize unligated junctions and direct MLH1-MLH3 to nick the opposite strand, analogous to the manner by which MSH2-MSH3 directs MLH1-MLH3 in MMR and in trinucleotide repeat expansion (Figure 2). Although this model benefits from parsimony in that MLH1-MLH3 need generate only two nicks rather than four, further research is necessary to confirm this hypothesis.

## 3. Independent Roles for MLH Proteins in Homologous Recombination and Meiosis

MLH factors have been thought to primarily function as heterodimers with MLH1 as the common binding partner. In this section, we will attempt to consolidate observations within the literature hinting at expanded roles for the MLH family, in addition to proposing explanations for these findings.

### 3.1. Human MLH3 and PMS2 (Homolog of Baker’s Yeast PMS1) Function Separately from MLH1 in Somatic Homologous Recombination

**Figure 4:**
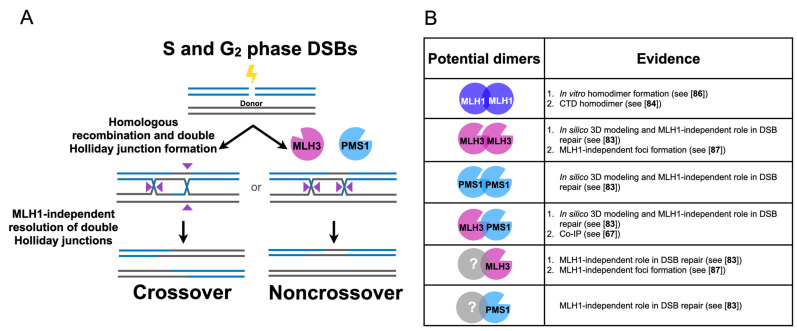
(**A**) MLH1- and MSH2-independent roles for MLH3 and PMS1 in mitotic homologous recombination. (**B**) Evidence for noncanonical MLH subunit interactions (see text for details).

The MLH family of proteins has not been extensively characterized for direct roles in somatic double-strand-break repair. In a recently published study, Rahman et al. [83] deleted *MLH1, PMS2, MLH3*, and *MSH2* in human TK6 B cells and found that PMS2 and MLH3 were important for the repair of double-strand breaks, whereas MLH1 and MSH2 appeared dispensable, providing evidence for roles in human cells for PMS2 and MLH3 independent of MMR (Figure 4A). Furthermore, PMS2 and MLH3 appeared to be involved in the resolution of double Holliday junctions as measured by the decrease in sister chromatid exchange events seen in *PMS2* and *MLH3* mutants. Curiously, PMS2 and MLH3 functions were each dependent on intact nuclease domains present in their C-terminal interaction domains [78,83,84,85]. Additionally, *PMS2* and *MLH3* gene disruptions were nonepistatic, suggesting that PMS2 and MLH3 act independently in double-strand-break repair. Whether a nuclease role is required in these MLH1-independent processes has yet to be confirmed biochemically, but the observation by Rahman et al. [83] that ectopic expression of the Holliday junction resolvase GEN1 (YEN1 in yeast) was sufficient to partially rescue the defects observed in the absence of PMS2 or MLH3 leaves open the possibility that the nuclease activities of PMS2 and MLH3 are involved in the direct processing of joint molecule intermediates, such as double Holliday junctions (Figure 4A).

How might PMS2 and MLH3 perform roles independent of MLH1? One of several possibilities (e.g., acting as monomers, interacting with other factors) is that PMS2 and MLH3 act as homodimers. Indeed, in silico analysis suggests that PMS2 and MLH3 are capable of homo/heterodimerization [83], and MLH3 has been observed to co-immunoprecipitate with PMS1 in meiotic budding yeast cells [67]. Furthermore, purified yeast MLH1 forms homodimers [86], and a homodimer of the C-terminal domain of yeast MLH1 has been crystallized (Figure 4B; see PBD 3RBN cited in [84]). If MLH factors exist separately from MLH1, and such complexes are functional in homologous recombination [83], it is conceivable that human PMS2/budding yeast PMS1 and MLH3 may also function independently of MLH1 in other processes, such as in meiosis. As discussed below, unexpected observations in the meiotic literature have been difficult to explain from the standpoint of canonical MLH complexes. By entertaining the possibility that MLH factors possess separable functions independently and in complex with MLH1, these enigmatic results may be easier to clarify.

### 3.2. Differential Timing and Location of Mouse MLH1 and MLH3 Foci Formation in Meiotic Recombination

**Figure 5:**
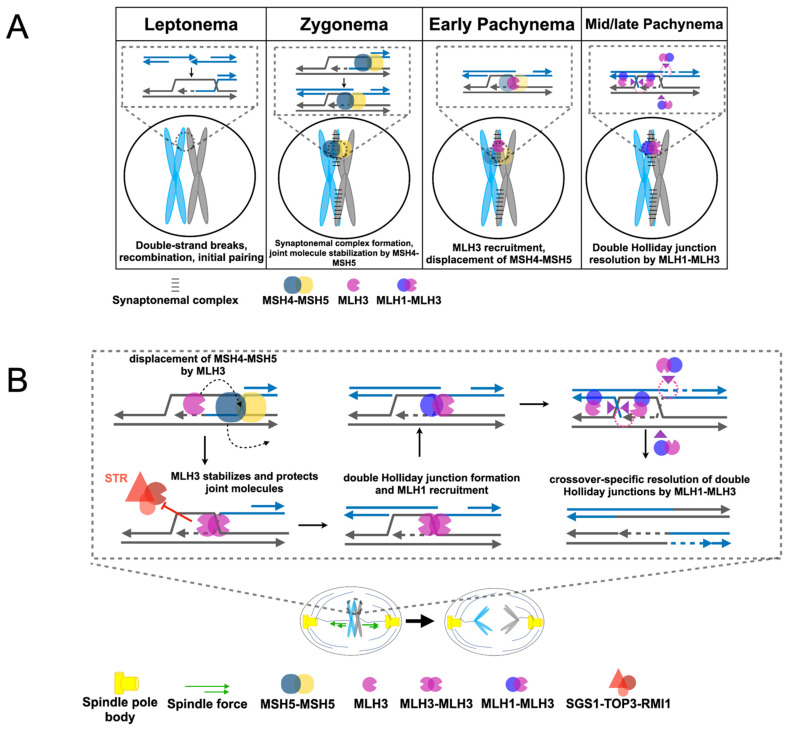
Models to explain the different meiosis I nondisjunction phenotypes of *mlh1*∆ and *mlh3*∆ mutants in meiosis. (**A**) Timeline of MSH4-MSH5, MLH3, and MLH1 foci formation on meiotic chromosomes. In leptonema, double-strand breaks and subsequent recombination drive initial pairing of homologous chromosomes. MSH4-MSH5 is recruited to recombination sites in zygonema, stabilizing single-end invasion intermediates. MLH3 foci formation in early pachynema is accompanied by decreased MSH4-MSH5 foci numbers. In mid-/late pachynema, MLH3 recruits MLH1 to form the MLH1-MLH3 complex that resolves double Holliday junctions to generate crossover products [87]. (**B**) A model for how MLH3 may promote CO-independent chromosome segregation. MLH3 is recruited to recombination intermediates, where it binds Holliday junctions, displacing MSH4-MSH5 and protecting nascent joint molecules from being unwound by the STR complex. These semi-stable joint molecules may provide sufficient tension to allow for biorientation of homologous chromosomes. MLH3 later recruits MLH1 to form the MLH1-MLH3 heterodimer.

Some of the earliest speculation of noncanonical MLH complexes in meiosis came from cytological work in mice in the early 2000s. Lipkin et al. [72] noticed the differential appearance of MLH1 and MLH3 foci in meiotic prophase in spermatocytes. They found that MLH3 localized to sites of recombination in early pachynema, but then colocalized with MLH1 in mid-pachynema. This MLH3 localization to recombination sites in early pachynema was independent of MLH1 (Figure 5A; [87]). MLH1 foci formation, however, was dependent on MLH3, indicating that MLH3 acts upstream of MLH1 and likely recruits MLH1 to CO-designated sites. Additionally, MLH3 formed foci at repetitive sequences at centromeres and on the Y chromosome independently of MLH1, but colocalized with MLH1 to these regions in a *Pms2^−/−^* background [87]. This localization of MLH3 to repetitive sequences was dependent on MSH2-MSH3, perhaps reflecting an intrinsic behavior of MLH complexes to interact with loop mismatches that form in repetitive DNA. Given that MLH1-MLH3 is known to be recruited by MSH2-MSH3 for MMR and trinucleotide repeat expansion [18,19,20], it may be that recruitment of MLH3 to repetitive sequences by MSH2-MSH3 is reflective of an intrinsic behavior of MLH complexes.

As indicated above, MLH3 localizes in mouse meiosis to repetitive sequences in a process dependent on MSH2-MSH3 but independent of MLH1. These observations were further complicated by the colocalization of MLH1 and MLH3 seen at repetitive sequences in *Pms2^−/−^* mice, indicating that deletion of a single MLH factor could lead to a redistribution of MLH factors throughout the genome (see discussion in [87] for further details). If MLH3 alone is playing a functional role, it must be doing so in a process that is distinct from MMR, trinucleotide repeat expansion, and meiotic crossover resolution, which all require intact MLH1-MLH3 complexes [20,21,34,83,85]. While difficult to interpret, these data hint at the possibility of MLH3 acting in an uncharacterized step in meiosis as a homodimer or through an unknown partner.

### 3.3. Differential Crossing Over and Spore Viability Phenotypes of MSH and MLH Mutants in Yeast Suggest CO-Independent Mechanisms of Chromosome Segregation

We have discussed the evidence in favor of a model in which MLH proteins possess functionality independently of MLH1. We will now look at genetic studies in yeast suggesting that the CO-promoting roles of MSH4-MSH5 and MLH1-MLH3 are separable from their roles in promoting accurate segregation of homologous chromosomes. Defects in the Class I CO-promoting machinery (e.g., mutations that disrupt ZMM, MLH1-MLH3, and EXO1 proteins), are correlated with low meiotic spore viability in baker’s yeast due to increased prevalence of MI-nondisjunction events where both homologs segregate toward the same pole during the first meiotic division [2,64,69,77,78,88,89,90]. Accordingly, Class I CO mutants in baker’s yeast exhibit the spore-viability pattern of 4, 2, 0 viable spores, indicative of frequent MI-nondisjunction. The elevated frequency of achiasmate homolog pairs in these mutants (homologs that fail to receive at least one CO), as well as the presence of randomly placed noninterfering COs, are thought to be the principal drivers of MI-nondisjunction.

Despite the well-established role for COs as the primary providers of interhomolog linkage, other data paint a more complicated picture of homolog-segregation methods. Argueso et al. [77] and Nishant et al. [78] showed that baker’s yeast mutants defective in both interference-dependent Class I and interference-independent Class II CO resolution pathways (*mlh1**Δ** mms4*Δ** and *mlh3*Δ* mms4*Δ**) displayed 6 to 17-fold reductions in COs. Curiously, other combinations of Class I and Class II mutants (*msh5*Δ* mms4*Δ** and *msh5*Δ* mms4*Δ* mlh1*Δ**) exhibited smaller defects (4 to 6-fold reductions) in crossing over than *mlh1*Δ* mms4*Δ** and *mlh3*Δ* mms4*Δ** mutants. Why might *msh5*Δ* mms4*Δ** and *msh5*Δ* mms4*Δ* mlh1*Δ** mutants show weaker CO defects, given that both Class I and Class II CO pathways are expected to be disrupted? One possibility is that the presence of MSH4-MSH5 obstructs alternative resolution pathways (i.e., SLX1-SLX4 or YEN1), thus accounting for the dramatic CO defect observed in *mlh1*Δ* mms4*Δ** and *mlh3*Δ* mms4*Δ** double mutants (see also [26]). Surprisingly, the spore viabilities of *mlh1*Δ* mms4*Δ** (42%) and *mlh3*Δ* mms4*Δ** (62%) double mutants were found to be relatively high compared to *msh5*Δ** (36%), *msh5*Δ* mlh1*Δ** (37%), and *msh5*Δ* mms4*Δ** (19%) mutants [26,77,78,89]. These results are consistent with MSH4-MSH5 acting upstream of MLH1-MLH3, as inferred from *msh5*Δ* mms4*Δ* mlh1*Δ** triple mutants mimicking *msh5*Δ* mms4*Δ** double mutants in terms of CO defects. However, the differential relationship between spore viability and crossing over exhibited by these mutants is inconsistent with the expectation that crossing over and spore-viability defects manifest from the same underlying mechanistic failure. Rather, the presence of MSH4-MSH5 appears to be refractory to chromosome segregation defects, independently of its pro-crossover role.

By extrapolating the CO frequency observed over the tested interval on chromosome XV, Argueso et al. [77] and Brown et al. [89] calculated that *mlh1*Δ* mms4*Δ** or *mlh3*Δ* mms4*Δ** meiotic yeast cells would each receive roughly 6–7 COs, well below the minimum threshold of 16 needed for each chromosome to receive at least one CO. Although the exact numbers of COs per cell may differ greatly from this extrapolation, these results are inconsistent with the notion that COs are required for proper meiosis I chromosome segregation in yeast. Indeed, CO-independent means of chromosome segregation have been well documented in other species, primarily through centromeric pairing and/or pairing at repetitive sequences (reviewed in [50]). Together, these results suggest that CO-independent homolog segregation mechanisms exist in baker’s yeast, and in some manner may be related to the action of MSH4-MSH5.

### 3.4. Contrasting Meiotic Phenotypes for MLH1 and MLH3 in Yeast Hint at an Alternative Role for MLH3 in Meiosis

Claeys, Bouuaert, and Keeney [26] performed a structure-function analysis of MLH1-MLH3 in baker’s yeast and concluded that MLH1-MLH3 has a separable DNA-binding activity for Holliday junctions and single-stranded DNA. Residues required for binding to both DNA substrates mapped to the N-termini and linker domains of MLH1 and MLH3. They further noted that the linker regions of MLH1 and MLH3 were particularly important for Holliday junction binding. The authors observed that *mlh3*∆ mutants exhibited a higher frequency of MI-nondisjunctions compared to *mlh1*∆ mutants. This result was unexpected because prior studies indicated that *mlh1*∆ mutants displayed spore viabilities less than or equal to *mlh3*∆ mutants, and *mlh1*∆ and *mlh3*∆ mutants displayed similar decreases in crossing over [26]. Curiously, they noted that *mlh1* N-terminal mutants mirrored the phenotype of *mlh3*∆ mutants, whereas *mlh3* linker mutants closely resembled *mlh1*∆. The authors proposed a number of possible explanations for this apparent paradox between MI-nondisjunction and spore viability phenotypes. First, it could be that the assayed chromosome (chromosome VIII) is nonrepresentative of global disjunction among all 16 chromosomes. It is known that chromosome size impacts the density of double-strand-break hotspots, crossover rates, and interference [69,91,92,93,94]. Alternatively, the spore viability defect in *mlh1*∆ is due to moderate MI-nondisjunction due to CO defects plus haplolethal mutations that arise during replication resulting from the absence of MLH1-PMS1. Conversely, the spore-viability defect observed in *mlh3*∆ cells may be entirely due to excess MI-nondisjunction.

Importantly, these hypotheses only explain why the differential nondisjunction phenotypes of *mlh1* and *mlh3* yeast mutants were not seen in prior studies of spore viability. To explain why these phenotypes manifest, Claeys, Bouuaert, and Keeney [26] propose that in *mlh1*∆ and *mlh3* linker mutants, joint molecules remain long enough to promote biorientation of homologs, but are ultimately resolved by structure-selective nucleases into a mixture of COs and NCOs or disassembled by STR. In this model, *mlh3*∆ and *mlh1* N-terminal mutants are unable to stabilize early recombination intermediates, thus experiencing higher levels of chromosome mis-segregation.

## 4. MLH3 may Promote Meiotic Homolog Disjunction through a Crossover-Independent Mechanism

To understand the above observations, we hypothesize that MSH4-MSH5 and MLH3 cooperate in an early stage of meiotic recombination to stabilize Holliday junction intermediates. In this model, accurate chromosome segregation is promoted by two distinct mechanisms: joint molecule stabilization and CO formation. First, Holliday junction binding by MSH4-MSH5 and other ZMMs provides a semistable intermediate to which MLH3 alone is recruited. We view this as being analogous to the role for stabilizing intermediates in somatic recombination that could explain observations made in Rahman et al. [83]. Once recruited, MLH3 may displace MSH4-MSH5 and bind stably to Holliday junctions, protecting them from disassembly by STR (Figure 5B). In support of this idea, studies in *Sordaria* and mice have suggested that MSH4-MSH5 foci tend to diminish around early to mid-pachynema, the same timeframe in which MLH3 foci (early pachynema) and MLH1 foci (mid-pachynema) are observed to form [87,95,96] (Figure 5A). It has also been proposed that the relative binding affinities of MLH1-MLH3 and MSH4-MSH5 for branched DNA substrates is consistent with MSH4-MSH5 displacement by MLH1-MLH3 [26,29,30,97,98]. This hypothesis is also consistent with studies in mice suggesting *mlh3*∆ and nuclease-dead *mlh3* mutants exhibit increased BLM (SGS1 in yeast) foci formation in zygonema [73,99], an earlier timepoint than when MLH3 is known to function.

If MLH3 is acting as a Holliday junction stabilizer, it would be expected to block Holliday junction disassembly by STR. Indeed, MLH3 coimmunoprecipitates with SGS1 in meiotic yeast cells [62], and *mlh3*∆ mutants show global increases in NCOs [85], possibly through excessive SGS1-mediated, synthesis-dependent strand annealing. It is possible this latter observation could be explained by alternate double Holliday junction resolution pathways involving structure-selective nucleases. However, work by Arter et al. [100] supports a model in which at least one structure-selective nuclease in budding yeast, YEN1, when ectopically expressed in pachytene, is capable of resolving ZMM-stabilized double Holliday junctions exclusively into COs, even in the absence of MLH1-MLH3 [100]. Whether MUS81-MMS4 or SLX1-SLX4 are capable of CO-specific resolution of double Holliday junctions in budding yeast remains unclear, although fission yeast and *C. elegans* rely on these structure-selective nucleases for Class I COs (reviewed in [49]). Together, these results are consistent with MLH3 acting to stabilize Holliday junctions. Furthermore, a recent IP-mass spectrometry screen and MLH3 pull-down experiments failed to detect any interaction between SGS1 and MLH3 at times just prior to and after CO formation [67,80], suggesting the SGS1-MLH3 interaction occurs either early in meiotic recombination or transiently at a later timepoint.

In summation, this model predicts a transient MLH1-independent role for MLH3 in stabilizing early recombination intermediates, thus promoting accurate chromosome segregation via a mechanism separate from its late role in double Holliday junction resolution. Such a model is consistent with studies of MLH2, suggesting it functions with MLH1 to limit the extent of heteroduplex tract lengths [4]. Deletion of *MLH2* leads to increases in spore viability of ZMM mutants *zip4∆* and *msh4∆* without increasing overall CO numbers, presumably due to longer heteroduplexes counterbalancing the joint molecule stabilization lost in the absence of ZMMs [3,4]. The contrasting MI-nondisjunction phenotypes of *MLH1* and *MLH3* mutants may thus be explained as follows. In *mlh1*∆ mutants, MLH3 is free to displace MSH4-MSH5 and stabilize Holliday junctions, while the absence of MLH1-MLH2 results in more stable heteroduplexes. Similarly, *mlh3*-linker mutants experience only moderate levels of MI-nondisjunction because *mlh3*-linker mutants are defective for displacement of MSH4-MSH5 due to defects in Holliday junction binding. In these mutants, MSH4-MSH5 may remain bound to Holliday junctions, acting to biorient homologs. Also, the presence of *mlh3*-linker mutants may sequester a portion of MLH1, keeping MLH1-MLH2 levels in check. Contrastingly, in the absence of MLH3, a portion of the MLH1 pool may bind to MLH2, limiting the extent of gene conversion tracts, thus destabilizing recombination intermediates, all while lacking the pro-Holliday junction stabilization provided by MLH3, culminating in high meiosis I nondisjunction levels.

## 5. Conclusions and Future Directions

In this review, we first presented the current state of the literature concerning the known roles for MLH factors in various aspects DNA metabolism. We then proposed a model to explain the numerous enigmatic MLH mutant phenotypes spanning over two decades. Namely, we proposed that MLH3 functions independently from MLH1, acting early in meiosis to stabilize nascent Holliday junctions, simultaneously promoting CO-independent homolog stabilization, in addition to directing recombination intermediates down to the Class I interfering CO pathway. We supported this hypothesis with data from several model organisms encompassing an amalgamation of cytological, genetic, and biochemical analyses. Our goal was to encourage research and promote speculation into rethinking how the MLH proteins are thought to function. As discussed in prior sections, the recent confirmation that human PMS2 and MLH3 not only function in somatic homologous recombination, a new role for MLH proteins, but do so independently of MSH2-MSH6, MSH2-MSH3, and MLH1, begs the question as to whether MLH proteins possess the ability to homodimerize, heterodimerize with unidentified partners, and perform biologically relevant functions via hitherto unexplored mechanisms. We present one such model that is readily testable. For example, this model predicts physical interactions with early meiotic factors that could be detectable in vitro or in vivo by immunoblot assays. Additionally, in vitro biochemistry using purified MLH factors in noncanonical combinations may elucidate new functionality (see [4]). Taken together, these results paint an exciting and relatively unexplored picture of MLH proteins. We hope this review will encourage further research exploring noncanonical roles for MLH complexes in somatic as well as meiotic cells.

## Data Availability

No new data were created or analyzed in this study. Data sharing is not applicable to this article.
